# 2378: A scientometric analysis of CTSA collaboration and impact

**DOI:** 10.1017/cts.2017.72

**Published:** 2018-05-10

**Authors:** Kristi Holmes, Ehsan Mohammadi, Karen Gutzman, Pamela Shaw, Donald Lloyd-Jones

## Abstract

OBJECTIVES/SPECIFIC AIMS: Translational science supports the continuum of activities from early-stage bench research to implementation of discoveries for better and faster treatments to more patients. Past studies have attempted to clarify our understanding of the spectrum of translational research by categorizing the activities into stages ranging from T0 to T4 using explanatory definitions. Unfortunately, this approach is often vague and relies on a process of manual classification and binning of research publications into predetermined categories. This study aims to provide a big-picture analysis of clinical and translational science (CTS) based on an in-depth analysis of the entire corpus of publications resulting from research funded by Clinical and Translational Science Awards (CTSA) U54 awards (through 2016). METHODS/STUDY POPULATION: We harvested bibliographic metadata from all papers that cited any of the U54 award numbers since the inception of the CTSA program to the most recent award announcement. Natural language processing techniques were used to create term co-occurrence networks based on English-language textual data. Relevant and nonrelevant terms were distinguished algorithmically and processed accordingly to provide the clustered visualization. RESULTS/ANTICIPATED RESULTS: With this approach, we uncovered 6 natural clustered areas of emphasis of published CTS research, the evolution of specific concepts through time, and gained a better understanding of their relative impact as demonstrated by citations. We performed additional analyses including discipline-specific impact assessment; identification of categories of excellence relating to both productivity and citations; characteristics of collaborative networks such as organizational, industry, and international collaborations and network dynamics; and resulting global impact of the CTSA program. DISCUSSION/SIGNIFICANCE OF IMPACT: Ultimately we gained a clearer understanding of the CTSA program, its evolution through scholarly publications, and key areas of impact of the program using computational, data-driven evaluation methods.

